# Emergence of Toscana Virus, Romania, 2017–2018

**DOI:** 10.3201/eid2705.204598

**Published:** 2021-05

**Authors:** Corneliu P. Popescu, Ani I. Cotar, Sorin Dinu, Mihaela Zaharia, Gratiela Tardei, Emanoil Ceausu, Daniela Badescu, Simona Ruta, Cornelia S. Ceianu, Simin A. Florescu

**Affiliations:** Dr. Victor Babes Clinical Hospital of Infectious and Tropical Diseases, Bucharest, Romania (C.P. Popescu, M. Zaharia, G. Tardei, E. Ceausu, S.A. Florescu);; European Society of Clinical Microbiology and Infectious Diseases Study Group for Infectious Diseases of the Brain, Basel, Switzerland (C.P. Popescu, M. Zaharia);; Carol Davila University of Medicine and Pharmacy, Bucharest (C.P. Popescu, M. Zaharia, S. Ruta, S.A. Florescu);; Cantacuzino National Medico–Military Institute for Research and Development, Bucharest (A.I. Cotar, S. Dinu, D. Badescu, C.S. Ceianu);; Stefan S. Nicolau Institute of Virology, Bucharest (S. Ruta)

**Keywords:** Toscana virus, *Toscana phlebovirus*, TOSV, *Phlebovirus*, *Phenuiviridae*, encephalitis, adults, phylogenetic analysis, viruses, Romania

## Abstract

We describe a series of severe neuroinvasive infections caused by Toscana virus, identified by real-time reverse transcription PCR testing, in 8 hospitalized patients in Bucharest, Romania, during the summer seasons of 2017 and 2018. Of 8 patients, 5 died. Sequencing showed that the circulating virus belonged to lineage A.

*Toscana phlebovirus* (TOSV; genus *Phlebovirus*, family *Phenuiviridae*) is transmitted by sand flies. Three genetic lineages (A, B, and C) with different geographic distribution have been described to date. TOSV is the only sand fly–transmitted virus causing neuroinvasive disease in humans and the most prevalent arthropodborne virus in the Mediterranean area; however, it remains a neglected pathogen and is seldom included in the diagnostic algorithm for central nervous system (CNS) infections (*1*–*4*).

An increased number of acute viral CNS infections were reported during the summer and fall seasons during 2016–2018 in Romania. Many of them, including severe cases, were confirmed as West Nile virus (WNV) infections; additional cases were caused by herpes and enteroviruses infections (*5*). Nevertheless, several severe cases, diagnosed mainly in elderly patients, remained without a known etiology. We describe the evidence of TOSV involvement in these neuroinvasive infections in patients admitted to a tertiary-care facility (Dr. Victor Babes Clinical Hospital of Infectious Diseases, Bucharest, Romania).

## The Study

We tested 31 adult patients (18 in 2017 and 13 in 2018) with neurologic manifestations; all tested negative by cerebrospinal fluid nucleic acid testing for WNV, herpesviruses, and enteroviruses. Seven confirmed cases and 1 probable case of TOSV neuroinvasive disease were identified by real-time reverse transcription PCR (rRT-PCR); cycle threshold values ranged from 34.61 to 41.18.

All cases were characterized by progression to severe illness (encephalitis in 7 cases and meningoencephalitis in 1 case). Cerebrospinal fluid (CSF) was analyzed after lumbar puncture in all patients. Computed tomography of the brain was performed in 7 cases, and cerebral magnetic resonance imaging was performed in 1 case.

Median age of patients was 77.75 years (range 68–91 years); 5 were men, and 3 were women. Underlying conditions were recorded in all patients, most frequently hypertension (5 cases), diabetes mellitus and ischemic heart disease (3 cases), and stroke sequelae and congestive heart failure (2 cases). Five patients died, 2 recovered with sequelae, and 1 had complete recovery.

Although this is a retrospective study, informed consent was obtained from each patient included in the study as part of the routine hospital activity. Demographic data, clinical features, diagnostics and outcome of patients are summarized in Appendix Table .

In all patients TOSV RNA was detected by using a TaqMan assay. Standard nested PCRs for large and medium segments were negative for all tested samples. In the 7 confirmed cases, both CSF and serum samples collected 1–4 days after illness onset were positive and the rRT-PCR amplicons were sequenced. In the probable case, only the serum sample was positive, but no sequence could be obtained. Urine samples collected on day 3 after illness onset in 2 patients were also positive by rRT-PCR.

On the basis of the short sequence of the small genomic segment derived from our patient samples, we determined that the virus belongs to genetic lineage A. The sequences were deposited in the European Nucleotide Archive (accession nos. LR735597–603) (Figure)

**Figure:**
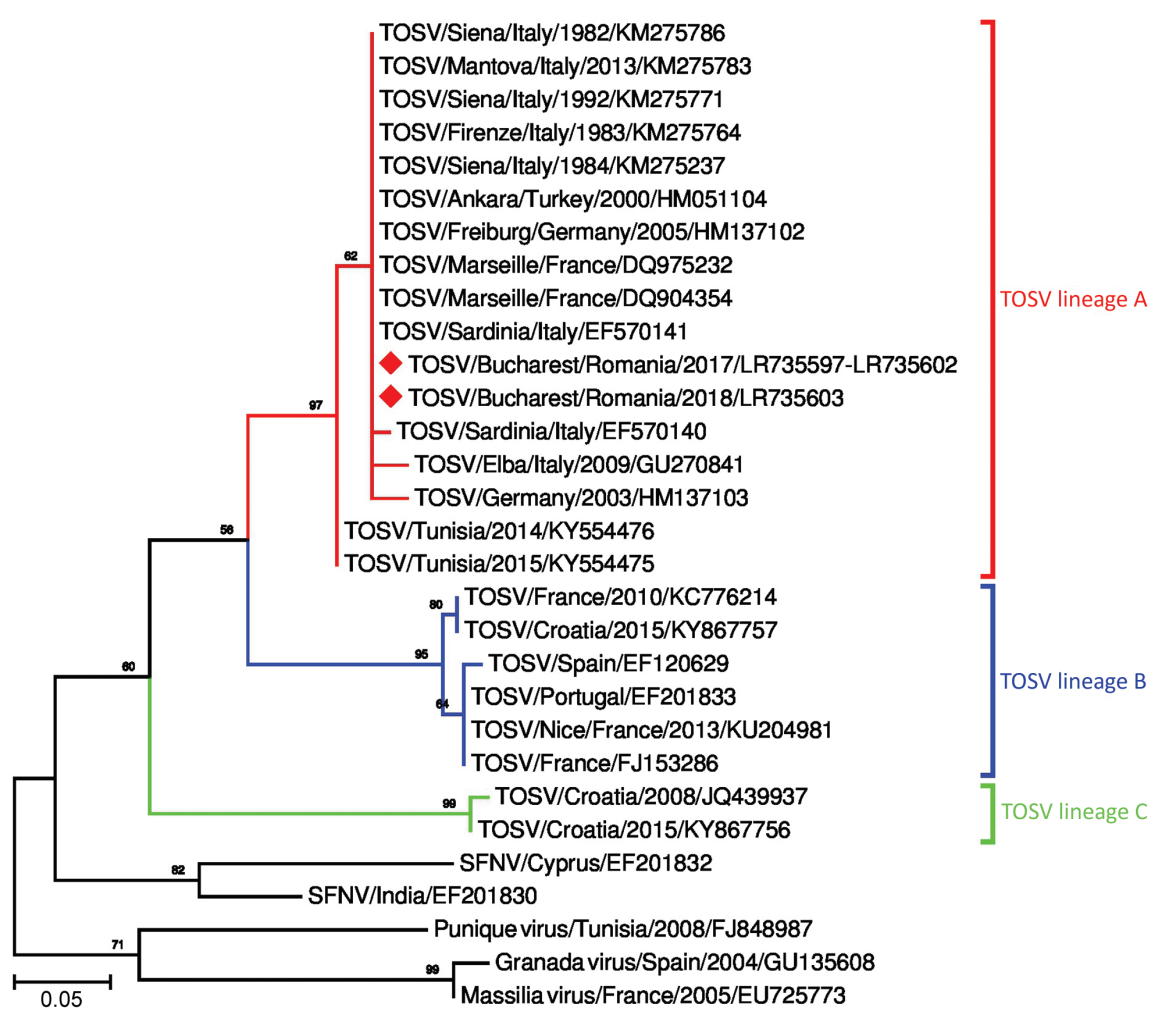
Phylogenetic tree of TOSV identified in 8 patients, Bucharest and surrounding area, Romania, 2017–2018, and reference sequences. Red diamonds indicate sequence obtained in this study; the other sequences included in the analysis were retrieved from GenBank. Numbers at nodes represent bootstrap percentages (values <50% are not shown). Phylogenetic relatedness was inferred from a 111-nt sequence of nucleocapsid gene, small segment (positions 1392–1502, numbering according to GenBank accession no. NC_006318.1) by using the neighbor-joining, Kimura 2-parameter method and 1,000 bootstrap replicates. SFNS, sandfly fever Naples virus; TOSV, Toscana virus.

The serologic tests performed poorly in these patients; IgM was detected in only 1 patient by indirect immunofluorescence test at the lowest dilution of 1:10. In 2 convalescent patients, seroconversion for IgG was found in samples collected 28 days apart by indirect immunofluorescence test in 1 patient and by immunoblotting in both patients.

## Conclusions

We describe 8 cases of CNS infections with TOSV, all in elderly patients, 7 of whom were residents of the city of Bucharest and 1 of the surrounding county (Ilfov). The onset dates ranged from June 17–September 1, overlapping the transmission period of WNV in the Bucharest area. Simultaneous occurrence of cases of vectorborne WNV and TOSV CNS infections were previously reported in southeastern Europe (*6*). Progression to severe illness might be linked to older age, and this observation might be biased by the selection of cases referred to a tertiary-care hospital with an intensive care unit. TOSV has been previously associated with human neurologic infections, ranging from mild disease to severe cases, both in the autochthonous population and in travelers, but with a low mortality rate (*1*,*7*,*8*). A case of severe meningoencephalitis was previously reported in a brother and sister, both of whom recovered but had neurologic sequelae (*9*). Other neurologic manifestations such as Guillain-Barré syndrome, polymyeloradiculopathy, hydrocephalus, change of personality, or hearing loss have been described (*4*).

In our study, the diagnosis relied on rRT-PCR detection of the viral RNA, followed by amplicon sequencing, because very few samples showed serologic reactivity. Negative results obtained when the samples were tested by using standard nested PCRs for large and medium genes can be explained by the low viral load, as indicated by the high cycle threshold values (34.61–41.18) in rRT-PCR tests. Viral RNA was detected only in serum and CSF samples collected during the first 1–4 days after illness onset; urine proved to be a valuable specimen for molecular diagnosis, as previously reported (*10*).

The reason for the poor performance of serologic assays in our case series is not clear but might be related to the patients’ immune status. An enzyme immunoassay test did not demonstrate high sensitivity, as previously reported, and the average percentage agreement between the commercial assays we used was low (57.8%), an observation also made by other researchers (*10*,*11*). On the other hand, all of our patients had an abrupt onset of symptoms, including rapid progression and hospitalization (median 2.4 days from onset, range 1–5 days). Sampling took place very early after illness onset, when antibodies levels are low and difficult to detect, possibly before seroconversion. The rapid death of 5 of the 8 patients precluded longitudinal antibody testing. In these cases, detection of TOSV RNA by using rRT-PCR, now the reference standard for TOSV diagnosis (*2*), was of paramount importance.

Genetic analysis based on a highly conserved 111-nt sequence showed that the circulating virus in Romania belonged to lineage A. This lineage has been described in Italy and southern France, in northern Africa (Tunisia), and in central and northern Anatolia (Turkey), but to our knowledge had never before recorded in southeastern Europe. Lineage B genotype has been reported in Spain, France, Portugal, Croatia, Morocco, and Turkey (*4*). A new genetic lineage of TOSV (lineage C) has been detected in Croatia, where it was co-circulating with lineage B TOSV (*12*). A novel variant of TOSV most closely related to lineage C has been detected in Greece (*7*,*13*). Other co-circulation of different lineages has been reported France and Turkey (lineages A and B) (*4*,*6*). No differences have been observed in the clinical picture or disease severity associated with these TOSV genotypes (*1*,*4*).

The emergence of TOSV in an urban area in southeastern Romania warrants attention to the sand fly vector. During 1939–1952, according to clinical records, sand fly viruses causing sandfly fever (i.e., 3-day fever or pappataci fever), transmitted by *Phlebotomus papatasi* sandflies, were thought to be circulating in southern Romania, with outbreaks occurring during the summer months. Bucharest and the surrounding Ilfov County area were thought to have been affected during 1944–1946 (*14*).

No recent data on sand flies are available for urban Bucharest and its surrounding area. During recent years, the distribution of some *Phlebotomus* sand fly species harboring TOSV was updated for Romania, including *P. perfiliewi*, *P. neglectus*, *P. sergenti*, but not *P. perniciosus* (*15*). Given that only a handful of severe cases were diagnosed at a tertiary-care hospital, the real magnitude of TOSV human infections and those of affected areas are unknown and warrant further study.

Appendix Additional information about emergence of Toscana virus, Romania, 2017–2018.
