# Supplementary material for: Emergence of Toscana Virus, Romania, 2017–2018
Source: Emerg Infect Dis. 2021 May;27(5):1482–5. doi: 10.3201/eid2705.204598 (PMC8084517; doi:10.3201/eid2705.204598)
Supplement: Appendix — Additional information about emergence of Toscana virus, Romania, 2017–2018. [file 20-4598-Techapp-s1.pdf]

Article DOI: https://doi.org/10.3201/eid2705.204598

Emergence of Toscana Virus, Romania, 2017–2018

Appendix

Methods

Case Definition

For encephalitis diagnosis the case definition from the 2013 Consensus Statement of the International Encephalitis Consortium was used. The major criterion was altered mental status (defined as decreased or altered level of consciousness, lethargy, or personality change) lasting ≥24 hours, with no alternative cause identified. Minor criteria were fever (temperature ≥38°C), generalized or partial seizures, new onset of focal neurologic findings, CSF leukocyte count ≥5 cells/mm^3, and abnormality of brain parenchyma on neuroimaging suggestive of encephalitis (*1*).

The main laboratory criterion was direct diagnosis of TOSV RNA, using real-time RT-PCR, a reference test for diagnostic purpose (*2*), followed by genomic sequencing. A positive sample by real-time RT-PCR, that could not be sequenced was defined as a probable case (*3*).

Laboratory Investigation

Paired samples of cerebrospinal fluid (CSF) and serum were collected from all patients at admission; a urine sample and a late serum sample at 28 days post-onset were also available for two patients.

Molecular Diagnosis

Molecular diagnosis was performed following Weidmann et al (*4*) protocol based on the primers and TaqMan probe for detection of TOSV targeting the S segment of the viral genome. RNA was extracted from different clinical samples (CSF, serum, plasma and urine) of suspected patients using QIamp viral RNA Mini kit (Qiagen, Hilden, Germany), according to the manufacturer’s instructions. The viral RNA was reverse transcribed and amplified using QuantiTect Probe RT-PCR kit (Qiagen, Germany) on Stratagene Mx3005P Real-Time PCR System. The following thermal profile was used: RT was performed at 52°C/30 min, followed by

polymerase activation at 95°C/15 min, 45 cycles of PCR at 94°C/15 s, 60°C/60 s. The primers and probe were used at final concentration of 0.36 μM, respectively 0.18 μM. Non template control used was nuclease free water, being included in each PCR run for validation. The standard nested PCRs for L and M segment were also performed (*5*).

Real-Time RT-PCR amplicons were purified using AmPure XP paramagnetic beads (Beckman Coulter) and sequenced with BigDye Terminator v3.1 Cycle Sequencing Kit on a SeqStudio Genetic Analyzer System (Applied Biosystems). Raw sequences were visually inspected, edited and their primer binding sites were removed with BioEdit version 7.0.5.3 (*6*). Phylogeny was inferred with Mega7 (*7*) from a DNA fragment spanning positions 1392–1502 (numbering according to NC_006318.1) of nucleocapsid gene, segment S.

Antibodies

Antibodies to sandflies viruses were tested with commercial kits. For screening, indirect immunofluorescence test (IIFT) for Sandfly fever virus Mosaic types Sicilian, Naples, Toscana, Cyprus IgM and IgG (Euroimmun, Luebeck, Germany) were used in 2017, and the EIA test Enzywell Toscana virus IgG/IgM (Diesse Diagnostica, Siena, Italy) was used in 2018. Immunoblotting assay *recom*Line HantaPlus IgG and IgM Strip-Immunoassay (Mikrogen Diagnostik, Neuried, Germany), with antigens produced by recombinant techniques, for the detection of IgG and IgM antibodies against various Hantaviruses, as well as Sandfly Fever Viruses (SFFV) serotypes Toscana and Sicilian, was used as a confirmatory support test.

References

1. Ayhan N, Charrel RN. An update on Toscana virus distribution, genetics, medical and diagnostic aspects. Clin Microbiol Infect. 2020;26:1017–23. PubMed https://doi.org/10.1016/j.cmi.2019.12.015

2. Venkatesan A, Tunkel AR, Bloch KC, Lauring AS, Sejvar J, Bitnun A, et al.; International Encephalitis Consortium. Case definitions, diagnostic algorithms, and priorities in encephalitis: consensus statement of the international encephalitis consortium. Clin Infect Dis. 2013;57:1114–28. PubMed https://doi.org/10.1093/cid/cit458

3. Centers for Disease Control and Prevention. Arboviral diseases, neuroinvasive and non-neuroinvasive

2015 case definition [cited 2020 Nov 10]. https://wwwn.cdc.gov/nndss/conditions/arboviral-diseases-neuroinvasive-and-non-neuroinvasive/case-definition/2015

4. Weidmann M, Sanchez-Seco MP, Sall AA, Ly PO, Thiongane Y, Lô MM, et al. Rapid detection of important human pathogenic Phleboviruses. J Clin Virol. 2008;41:138–42. PubMed https://doi.org/10.1016/j.jcv.2007.10.001

5. Sánchez-Seco MP, Echevarría JM, Hernández L, Estévez D, Navarro-Marí JM, Tenorio A. Detection and identification of Toscana and other phleboviruses by RT-nested-PCR assays with degenerated primers. J Med Virol. 2003;71:140–9. PubMed https://doi.org/10.1002/jmv.10465

6. Hall TA. BioEdit: a user-friendly biological sequence alignment editor and analysis program for Windows 95/98/NT. Nucleic Acids Symp Ser. 1999;41:95–8.

7. Tamura K, Dudley J, Nei M, Kumar S. MEGA4: Molecular Evolutionary Genetics Analysis (MEGA) software version 4.0. Mol Biol Evol. 2007;24:1596–9. PubMed https://doi.org/10.1093/molbev/msm092

Appendix Table. Case series of Toscana virus infection in Romania 2017–18

No.	Case/year	Age	Onset/residence/ Lenght of stay	Sample/Day post onset	IgM	IgG	Real-time PCR TOSV (Ct)	Sequence	TOSV infection	Clinical findings/ Computed tomography/Cerebrospinal fluid	Comorbidities	Outcome
1	Case 1/2017, Encephalitis	91	June 17/ Ilfov county/10 d	Serum/Day 2<br>CSF/Day 2	Neg[1]<br>NT	Neg[1]<br>NT	39.14<br>34.61	No<br>Yes	Confirmed	Fever, psychomotor agitation, loss of consciousness, coma/Cerebral atrophy/CSF: normal	Hypertension, congestive heart failure, ischemic heart disease, stroke sequelae	Died
2	Case 2/2017, Encephalitis	68	August 12/Bucharest/4 d	Serum/Day 2<br>CSF/Day 2	Neg[1]<br>NT	Neg[1]<br>NT	37.26<br>36.96	Yes<br>Yes	Confirmed	Fever, loss of consciousness, confusion, drowsiness myoclonus, coma./Cerebral atrophy and occipital stroke sequelae; CSF: Mild increase in proteins	Hypertension, diabetes mellitus, obesity, Non-Hodgkin lymphoma	Died
3	Case 3/2017, Encephalitis	72	July 28/Bucharest/21 d	Serum/Day 3<br>CSF/Day 3	Neg[1]<br>NT	Neg[1]<br>NT	37.72<br>37.97	Yes<br>Yes	Confirmed	Fever, arthralgia, insomnia, aphasia, confusion, psychomotor agitation; CSF: mild increase in proteins, 6 leukocytes/mm^3	Hypertension	Complete resolution
4	Case 4 /2017, Encephalitis	79	August 2/Bucharest/19 d	Serum/Day 4<br>CSF/Day 4	Neg[1]<br>NT	Neg[1]<br>NT	41.13<br>38.47	Yes<br>Yes	Confirmed	Fever, confusion, aphasia/Normal/CSF: Mild increase in proteins	Diabetes mellitus type II Ischemic heart disease	Died
5	Case 5/2017, Meningoencephalitis	70	August 7/Bucharest/4 d	Serum/Day 4<br>CSF/Day 4	Pos [1]<br>1/10<br>NT	Neg[1]<br>NT	40.97<br>37.96	Yes<br>Yes	Confirmed	Fever, chills, headache, vomiting, confusion, coma/Normal/CSF: Elevated proteins, 68 leukocytes/mm^3	Diabetes mellitus type II, Ischemic heart disease, Atrial fibrillation, Congestive heart failure, Diabetic polyneuropathy and arteriopathy	Died
6	Case 6/2017, Encephalitis	85	August13/Bucharest/6 d	Serum/Day 1<br>CSF/Day 1	Neg[1]<br>NT	Neg[1]<br>NT	41.18<br>No Ct	No<br>-	Probable	Fever, confusion, coma/Normal/CSF: normal	Hypertension, Stroke sequelae, Chronic renal failure	Died
7	Case 7/2017, Encephalitis	78	August 24/Bucharest/12 d	Serum/Day 0<br>CSF/Day 0<br>Serum/Day 3<br>Urine/Day 3<br>Serum/Day 28	Neg[1, 3]<br>NT<br>Neg[1]<br>NT<br>Neg[1, 3]	Neg[1, 3]<br>NT<br>Neg[1]<br>NT<br>Pos[1,3]	36.05<br>38.42<br>Neg<br>36.29<br>NT	Yes<br>Yes<br>-<br>No<br>-	Confirmed	Fever, stiff neck, right hemiparesis, aphasia/Normal/CSF: Normal	Hypertension, Alzheimer disease	Recovery with sequelae

8	Case 8/ 2018, Encephalitis	79	August 15/Bucharest/32 d	Serum/Day 3	Neg[2, 3]	Border-line [2,3]	37.53	Yes	Confirmed	Fever, aphasia, tetraparesis, obtundation, vomiting, stiff neck, coma/Normal/CSF: 6 leukocyte cells/mm^3	Parkinson disease, Hypothyroidism	Recovery with sequelae
				Plasma/Day 3	NT	NT	36.50	Yes				
				CSF/Day 3	NT	NT	36.66	Yes				
				Urine/Day3	NT	NT	35.75	Yes				
				Serum/Day 10	Neg[3]	Border-line[3]	NT	-				
				Serum/Day 28	Neg[3]	Pos[3]	NT	-				

[1]IIFT; [2]ELISA, [3] Immunoblotting.
